# Integrative network analysis identifies cell-specific *trans* regulators of m^6^A

**DOI:** 10.1093/nar/gkz1206

**Published:** 2020-01-08

**Authors:** Sanqi An, Wanxu Huang, Xiang Huang, Yixian Cun, Weisheng Cheng, Xiang Sun, Zhijun Ren, Yaxin Chen, Wenfang Chen, Jinkai Wang

**Affiliations:** 1 Department of Medical Bioinformatics, Zhongshan School of Medicine, Sun Yat-sen University, Guangzhou 510080, China; 2 Center for Stem Cell Biology and Tissue Engineering, Key Laboratory for Stem Cells and Tissue Engineering, Ministry of Education, Sun Yat-sen University, Guangzhou 510080, China; 3 RNA Biomedical Institute, Sun Yat-sen Memorial Hospital, Sun Yat-sen University, Guangzhou 510120, China; 4 Center for Precision Medicine, Sun Yat-sen University, Guangzhou 510080, China

## Abstract

N6-methyladenosine (m^6^A) is a reversible and dynamic RNA modification in eukaryotes. However, how cells establish cell-specific m^6^A methylomes is still poorly understood. Here, we developed a computational framework to systematically identify cell-specific *trans* regulators of m^6^A through integrating gene expressions, binding targets and binding motifs of large number of RNA binding proteins (RBPs) with a co-methylation network constructed using large-scale m^6^A methylomes across diverse cell states. We applied the framework and successfully identified 32 high-confidence m^6^A regulators that modulated the variable m^6^A sites away from stop codons in a cell-specific manner. To validate them, we knocked down three regulators respectively and found two of them (TRA2A and CAPRIN1) selectively promoted the methylations of the m^6^A sites co-localized with their binding targets on RNAs through physical interactions with the m^6^A writers. Knockdown of *TRA2A* increased the stabilities of the RNAs with TRA2A bound near the m^6^A sites and decreased the viability of cells. The successful identification of m^6^A regulators demonstrates a powerful and widely applicable strategy to elucidate the cell-specific m^6^A regulators. Additionally, our discovery of pervasive *trans*-acting regulating of m^6^A provides novel insights into the mechanisms by which spatial and temporal dynamics of m^6^A methylomes are established.

## INTRODUCTION

N6-methyladenosine (m^6^A) is the most prevalent internal RNA modification in mRNA and long non-coding RNAs of eukaryotes. It is a reversible RNA modification prefers to occur on DRACH motif near stop codon and in long internal exon of mRNA (1,2). A nuclear methyltransferase complex consisting of METTL3, which is the catalytic subunit, METTL14, WTAP, VIRMA, ZC3H13, RBM15 (or RBM15B) and CBLL1/HAKAI catalyzes the m^6^A modifications co-transcriptionally, acting as m^6^A ‘writers’ (3–5). A specific m^6^A demethylase ALKBH5 as well as a less specific m^6^A demethylase FTO mediate the demethylation of m^6^As, acting as the m^6^A ‘erasers’ (6). A variety of proteins including YTH domain-containing proteins can specifically bind m^6^A marks as the m^6^A ‘readers’ and regulate a variety of post-transcriptional processes, such as RNA decay, alternative splicing, translation, alternative polyadenylation and nuclear export (7–9).

It is widely accepted that m^6^A RNA methylation is dynamically regulated (10). More and more studies reported the alterations of functionally important m^6^A sites caused by expression change of m^6^A writers and erasers played important roles in a variety of physiological and pathological processes (11–15). A recent study reported that 33–46% of the variability of m^6^A levels were due to *cis*-regulation, suggesting that the dynamics of m^6^A are likely through global regulation by modulating the abundances of methyltransferase components (16). However, it is still unclear whether *trans*-regulation plays important roles in site-specific dynamics of m^6^A levels. Besides global regulation, site-specific m^6^A dynamics can possibly be precisely established through the interplays of a variety of *trans*-acting m^6^A regulators with m^6^A writers and erasers at specific sites bound by the regulators. Indeed, m^6^A is deposited on nascent RNAs (17) and can be regulated co-transcriptionally through H3K36me3 histone modification (18) as well as transcription factors (19,20). Transcription factor CEBPZ recruits METTL3 to the promoter of a specific set of active genes to regulate the m^6^A of the associated mRNAs involved in acute myeloid leukemia (19). Similarly, transcription factors SMAD2/3 can selectively promote the m^6^A modifications of the genes involved in early cell fate decision through co-transcriptional recruitment of the m^6^A methyltransferase complex onto the nascent RNAs (20). On the other hand, Cao *et al.* reported two RNA binding proteins (RBPs) DDX46 and HNRNPA2B1 dynamically interacted with m^6^A erasers to regulate the m^6^A of genes critical for innate immunity in response to viral infection (21,22). Nevertheless, whether specific regulation of m^6^A is prevalent remains a mystery.

Systematical analyses of large-scale m^6^A methylomes are promising to elucidate the *trans*-acting m^6^A regulators. Theoretically, there should be a correlation between the gene expression of a *trans*-acting m^6^A regulator and the m^6^A levels of the m^6^A sites regulated by the regulator. However, in practice, it is very challenging due to various technical difficulties: (i) proper quantification of m^6^A is difficult due to various technical biases of m^6^A-seq data; (ii) it is almost impossible to afford serious multiple testing correction for massive correlation tests between genes and m^6^A sites; (iii) Pearson correlation of m^6^A levels quantified using m^6^A-seq suffers seriously by the outlier issue; (iv) correlations may not reflect direct effects of regulation.

In this study, we developed a computational framework to systematically identify cell-specific *trans* regulators of m^6^A through integrating gene expressions, binding targets and binding motifs of a large number of RBPs with a co-methylation network constructed using large-scale m^6^A methylomes across diverse cell states. We applied the framework to the public available m^6^A-seq data of 25 unique cell lines and successfully identified 32 high-confidence m^6^A regulators with reasonable experimental validation rate, demonstrating a powerful and widely applicable strategy to elucidate cell-specific the m^6^A regulators. Our discovery of pervasive *trans*-acting regulating of m^6^A provided novel insights into the mechanisms by which spatial and temporal dynamics of m^6^A methylomes were established.

## MATERIALS AND METHODS

### Processing of the m^6^A-seq data in multiple cell lines

Raw sequence data of 104 m^6^A-seq libraries (IP and input) from 25 unique cell lines were downloaded from Sequence Read Archive (SRA, https://www.ncbi.nlm.nih.gov/sra) (1–2,19,23–37). The accession numbers of these data can be found in Supplementary Table S1. The reads were mapped to hg19 human genome using HISTA2 (v2.1.0) (38). We used StringTie (v1.3.4d) (39) to calculate the TPMs (Transcripts Per Million) of Ensembl annotated genes using the input libraries, followed by quantile normalization of the TPMs across all samples. m^6^A peaks were identified according to the methods as described in our previous paper (23), which was modified from the method published earlier by Dominissini *et al.* (2). Briefly, we made sliding windows of 100 bp with 50 bp overlap on the exon regions and calculated the RPKM of each window. The sliding windows with winscore (enrichment score) >2 were identified as m^6^A peaks in each sample (2,23). To deal with the technical issue that lowly expressed windows might have unreliable winscores, we added 1 to the RPKM of each window in both IP and input before winscore calculation in order to penalize the windows with low RPKMs. We took the union of m^6^A peaks identified in these samples for further analyses. The m^6^A ratio of each peak was calculated as the RPKM (without adding 1) of IP library divided by the RPKM (without adding 1) of input library. To m^6^A ratios based on the denominators (peak RPKM of input) < 5 were treated as NAs (not available) in the downstream analyses. The m^6^A peaks with NAs in more than half of the samples were removed. The continuous m^6^A peaks in the same gene were merged, the merged peaks with more than 5 continuous sliding windows (300 bp) were then divided into multiple peaks that spanning no more than five sliding windows.

Different protocols of RNA fragmentations before immunoprecipitation in the preparations of different m^6^A-seq libraries might cause the variations of read signals at the actually same m^6^A peaks, resulting in diverse widths and centers of the actual same m^6^A peaks thus false m^6^A differences in certain regions, we therefore defined the m^6^A ratio of each merge peaks with multiple sliding windows as the maximum m^6^A ratio of all windows for each sample respectively. Global m^6^A differences among samples caused by diverse activities of m^6^A writers and erasers as well as technical variation of immunoprecipitation efficiencies would dilute and distort the signals of selective regulation of m^6^A, we therefore used quantile normalization to normalize the m^6^A ratios of the merged peaks across all samples.

### Analyses of the m^6^A ratios across multiple cell lines

Hierarchical clustering of all samples was performed using 1- Pearson correlation coefficient as distance metric based on m^6^A ratios or TPMs of the merged peaks with CVs > 0.7 or 1000 genes with the largest CVs. The two hierarchical clustering dendrograms were subsequently compared using the ‘dendextend’ package (40) implemented in R. HOMER software (41) was used for motif enrichment analysis using randomly permutated sequences as the backgroup for RNAs. To compare the overlaps of miCLIP-seq m^6^A sites (CITS + CIMS) in HEK293 cells (42) between stable m^6^A peaks and variable m^6^A peaks, we only used the m^6^A peaks identified in HEK293T cells according to the above-described pipeline. Distributions of m^6^A peaks were plotted on a mega gene with 10 bins in 5′ UTR, CDS, and 3′ UTR respectively using the methods as described in our previous paper (23). Radar plot was plotted using ‘fmsb’ package implemented in R.

### Construction of the co-methylation network

We merged the m^6^A ratios as well as TPMs of all samples from each of the 25 unique cell lines by taking the averages. 29173 m^6^A peaks with CV of m^6^A ratio across 25 unique cell lines >0.3 were used to construct the signed weighted m^6^A co-methylation network using WGCNA package (43) implemented in R. The adjacency matrix was constructed by raising the 0.5 + 0.5 × correlation matrix to the power of 7. The hierarchical clustering tree was then cut into 41 co-methylation modules using dynamic hybrid tree-cutting algorithm. The m^6^A index of each module was represented by the eigengene, which was the first component of Principal Component Analysis. The 41 modules were further clustered into 12 larger modules based on the correlation of their m^6^A indexes for the analyses required larger number of m^6^A peaks. Gene Ontology analysis was performed using DAVID with the genes in all the modules as the background (44).

### Analyses of the cancer module

The gene expression, mutation, and clinical data of TCGA (https://tcga-data.nci.nih.gov/) were downloaded from cBioPortal (45,46). We calculated the means of logarithm transformed TPM+1 of all genes in cancer the module as the gene expression index of the cancer module. We used the Cox regression to examine the correlations between gene expression indexes of the cancer module and patient survival in each cancer type. Gene Ontology analysis was performed using DAVID with the genes in all modules as the background (44).

### Identification of m^6^A regulators

We used 1442 expressed RBPs out of 1648 genes annotated under the term ‘RBP’ in Gene Ontology Database (47) to scan for m^6^A regulators by testing the Pearson correlation between the TPMs of RBPs and the eigengenes of the 41 m^6^A modules respectively. To exclude the spurious significances due to the outliers in Pearson correlation, for each correlation test between RBP expression and eigengene of co-methylation module, we used the maximum *P-*values of 25 Pearson correlations calculated based on 24 of the 25 cell lines (in the other word, we removed one cell line in each of the 25 correlation). The correlations with FDR < 0.2 were determined as significant correlations. The *P-*value cutoff corresponding to the FDR of 0.2 were determined based on the null distribution of *P-*values generated by 10 times permutations. In each permutation, we randomly relabeled the samples and performed the Pearson correlation between RBP expression and eigengene of co-methylation module using the above-described method. The *P*-value cutoff was further determined as the *P*-value under which the average number of significant correlations in permutations was only one fifth of the observed number of significant correlations using real data (Supplementary Figure S4A).

We downloaded the CLIP-seq peaks from starBase database (version 3) (48–50) as well as the ENCODE CLIP-seq dataset in HepG2 and K562 cells (51). Significance of the overlapping between a set of CLIP-seq peaks and an m^6^A module was calculated by testing whether the module and other modules (as background) had equal fraction of m^6^A peaks that overlap with the CLIP-seq peak (at least 1 bp) using χ^2^ tests. We obtained the 110 Motifs of 89 RBPs from a published dataset based on large scare in vitro RNAcompete (52) as well as other well-known RBP motifs (53–55). Significance of the enrichment of an RBP motif in an m^6^A module was calculated by testing whether the module and other modules (as background) had equal fraction of m^6^A peaks that contain the RBP motif using χ^2^ tests. The significant overlapping was defined as the ones with Benjamini-Hochberg FDR < 0.05 based on all the tests. The final list of m^6^A regulators were those RBPs with gene expression significantly correlated with the modules and with CLIP-seq targets or motifs significantly enriched in the same modules. The proteins that interact with METTL3, METT14, WTAP, VIRMA and m^6^A based on IP mass spectrum data were directly obtained from the published papers (4,56–59).

### Analyses of the low-input m^6^A-seq data

The reads of the second end were trimmed to 50 bp from 3′ end for downstream analyses. We mapped the raw data to human genome and calculate m^6^A ratios for each sliding window using the above-described protocol. We used ‘exomePeak’ package implemented in R to identify the m^6^A peaks and determine the differentially methylated m^6^A peaks with FDR < 0.05 (60). To examine whether the m^6^A peaks within the associated module or with CLIP-seq binding showed stronger switch of m^6^A ratios upon RBP knockdown, we calculated the fold change of m^6^A ratios upon RBP knockdown for each m^6^A peaks in all the modules. If one RBP significantly correlated with multiple modules as predicted, we merged these modules together as the RBP associated module for the analyses. To filter out the ambiguous fold change values due to small denominators, only the peaks with input window RPKM > 5 in all samples and m^6^A ratio > 0.1 in both replicates of control samples were considered for the analyses. The data were visualized using the Integrative Genomics Viewer (IGV) tool (61). Differential gene expression analyses were performed based on the input data using DESeq2 (62) according to the read counts of each gene determined by HTSeq (63). The genes with FDR < 0.05 and mean CPM (Couts per Million) > 100 were determined as the differentially expressed genes.

### Cell culture

Cells were maintained at 37°C with 5% CO_2_ in a humidified incubator and passaged every 2–3 days. Wild type HEK293T and HepG2 cells were cultured in high-glucose Dulbecco's Modified Eagle Medium (DMEM, ThermoFisher Scientific) supplemented with 10% FBS (ExCell Bio). All cells were tested for absence of mycoplasma contamination using Myco-Blue Mycoplasma Detector (Vazyme).

### Plasmid constructs and transfection

For gene knocking-down, short-hairpin RNA (shRNA) oligos were synthesized, annealed and inserted into pLKO.1 vector. The pLKO.1-shRNA plasmids were then transfected into HEK293T cells with packing vectors pMD2.G and psPAX2 to produce lentiviruses. To overexpress the RBPs, we inserted the full-length coding regions amplified from HEK293T cDNA library by polymerase chain reaction (PCR) into pCDNA3.1 vector followed by adding the Flag tag. The pCDNA3.1-RBP plasmids were transfected into HEK293T cells with Lipofectamine 2000 (Invitrogen) according to the manufacturer's instructions. All the sequences of shRNA oligos and PCR primers are listed in Supplementary Table S2.

### RNA isolation and real-time quantitative PCR

Total RNA was extracted using NucleoZOL reagent (Macherey-Nagel) or MiniBEST Universal RNA Extraction Kit (Takara, Japan). First-strand cDNA was synthesized by reverse transcription of 1 μg RNA using HiScript II 1st Strand cDNA Synthesis Kit (Vazyme, China) according to the manufacturer's protocol. Quantitative real time-PCR was performed using TB Green Premix Ex Taq (Takara, Japan) in QuantStudio 7 Flex Real-Time PCR System (Life Technologies, USA). *β-actin* and *GAPDH* were used as reference genes for input normalization. The mRNA expression was measured by quantitative PCR using the ΔΔCT method. Primers for quantitative PCR were listed in Supplementary Table S2.

### CRISPR-Cas9 mediated *METTL3* knockout

Transiently transfected plasmid expressing two sgRNAs targeting human METTL3 exons was adopted for internal fragment deletion in specified size according to CRISPR-Cas9-2hitKO system. Two target guide RNAs were designed using the online tool (http://tools.genome-engineering.org) with high scores and minor off-target effects and then subcloned into CRISPR-Cas9-2hitKO plasmid. (All the sequences of sgRNA oligos were listed in Supplementary Table S2). To establish the knockout cell lines, CRISPR-Cas9-2hitKO plasmid carrying two sgRNA-expressing cassettes were transfected into HepG2, GFP expressing cells were enriched by FACS (MoFlo Astrios EQ, Beckman Coulter) 3 days later and seeded at low density for single colony isolation. Knockout efficiency was tested by DNA sequencing and verified by western blotting.

### Co-immunoprecipitation and western blot

Whole-cell extracts were extracted by directly lysing the cells with 1 × RIPA Buffer (Cell Signaling Technology) with 1 mM PMSF (Beyotime) added immediately before use. Samples were boiled by adding 6 × sodium dodecyl sulphate (SDS) sample buffer for 10  min at 100°C and resolved using SDS-polyacrylamide gel electrophoresis. To perform immunoprecipitation, we lysed the cells by RIPA lysis buffer. The lysates were sonicated at 4°C and cleared by centrifugation at 12 000 rpm for 15  min at 4°C. Immunoprecipitation was carried out by incubating the FLAG beads (Bimake) at 4°C with the lysate overnight. Immunoprecipitates were washed three times in cold E1A lysis buffer (250 mM NaCl, 50 mM HEPES (pH 7.5), 0.1% NP-40, 5 mM EDTA, protease inhibitor cocktail (Roche)) and boiled with 2 × SDS sample buffer for 10 min. The proteins were probed with the following antibodies: METTL3 Rabbit mAb (1:2000, 15073-1-AP, Proteintech), METTL14 Rabbit mAb (1:500, 51104S, Cell Signaling Technology), Monoclonal ANTI-FLAG M2 antibody (1:1000, F1804, Sigma), GAPDH (1:2000, 5174, Cell Signaling Technology), ALKBH5 (1:3000, ab195377, abcam) and FTO (1:1000, 31687, Cell Signaling Technology). Immuno-detection was performed using HRP-conjugated Affinipure Goat Anti-Mouse IgG(H+L) (1:5000, SA00001-1, Proteintech) or HRP-conjugated Affinipure Goat Anti-Rabbit IgG(H+L) (1:5000, SA00001-2, Proteintech) and ECL prime substrate (Bio-Rad) according to the manufacturer's instructions.

### Low-input m^6^A-seq

Low-input m^6^A-seq was performed based on the protocols previously described by Zeng *et al.* (64) with several modifications. Briefly, a total volume of 8–10 μg total RNA was fragmented using the 10 × RNA Fragmentation Buffer (100 mM Tris–HCl, 100 mM ZnCl_2_ in nuclease free H_2_O) and purified with sodium acetate (Sigma-Aldrich), glycogen (Thermo Fisher Scientific) and 100% ethanol. A total of 30 μl of protein-A/G magnetic beads (10002D/10004D, Thermo Fisher Scientific) were washed twice in IP buffer (150 mM NaCl, 10 mM Tris–HCl, pH 7.5, 0.1% IGEPAL CA-630 in nuclease free H_2_O) and incubated with 5 μg anti-m^6^A antibody (202003, Synaptic Systems) in 500 μl of IP buffer at 4°C for at least 6 h. After washed twice in IP buffer, the antibody-bead mixture was resuspended by fragmented total RNAs in IP buffer and incubated at 4°C for 2 h. Then after washed twice in low-salt IP buffer, and twice in high-salt IP buffer at 4°C for 10 min each, the m^6^A enriched fragmented RNAs were eluted and purified from the beads using RNeasy Mini Kit (QIAGEN). Sequencing libraries were generated using the SMARTer Stranded Total RNA-Seq Kit v2 (634413, Takara). All libraries were sequenced on an Illumina HiSeq X Ten platform to produce 20–40 M strand-specific paired-end reads.

### mRNA stability assay

Cells were seeded into 6-well plates and treated with actinomycin D (5 mg/ml) for 4, 2 and 0 h after culturing for 12 h. We used NucleoZOL reagent (Macherey-Nagel) to exact the total RNAs followed by reverse transcription. The abundances of the interest genes were detected measured in each time point by real-time quantitative PCR (qPCR) using 18S rRNA as the reference gene. The qPCR primers are listed in Supplementary Table S2.

### Colony formation assay

After trypsinization and cell counting, 1200 HepG2 cells were seeded per well in 6-well plates and cultured in DMEM supplemented with 10% fetal bovine serum (FBS) for 7 days. Cells were rinsed with phosphate buffered saline once and fixed using paraformaldehyde and stained using crystal violet.

## RESULTS

### Development of a computational framework to systematically identify cell-specific m^6^A regulators

**Figure 1.**
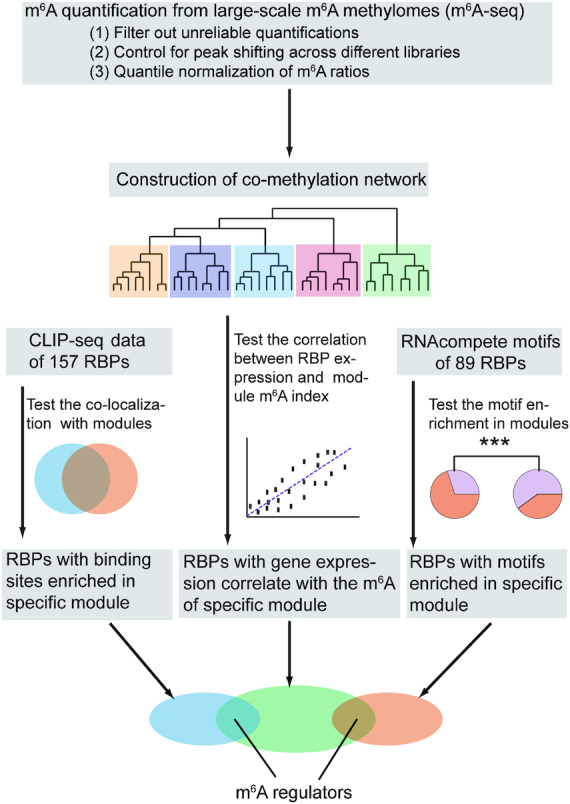
Schematic flow chart demonstrating the computational framework to identify *trans* regulators of m^6^A.

To overcome the above-mentioned difficulties of identifying cell-specific m^6^A regulators using large-scale m^6^A methylomes, we developed a computational framework through integrating gene expressions, binding targets and binding motifs of a large number of RBPs with a co-methylation network constructed using large-scale m^6^A methylomes across diverse cell states (Figure 1).

First of all, a variety of technical issues of m^6^A-seq data could hinder the successful systematic analyses of the quantitative m^6^A ratios calculated based on the m^6^A-seq data. Therefore, we performed multiple processes to minimize the influences of different types of technical issues (see ‘Materials and Methods’ section for details). Besides applying stringent filters to rule out the unreliable quantifications, we also merge the peaks across multiple samples and used the with the maximum m^6^A ratio of 100 bp window to represent the m^6^A ratio of merged peaks with continuous windows in each sample, therefore, the shifting of peak centers and divergence of peak breadths due to technical biases in preparing the m^6^A-seq libraries, such as variations in RNA fragmentation lengths and sequencing lengths, will be controlled. At last, quantile normalizations of the m^6^A ratios are performed, so that not only variations of antibody efficiencies can be corrected but also we can focus on capturing the mechanisms that regulate selective subsets of m^6^A peaks other than the global regulation dictated by m^6^A writers and erasers.

We hypothesis that the m^6^A sites regulated by the same m^6^A regulators should have correlated m^6^A levels (co-methylation) across different cell states, and the m^6^A levels of the module should also be correlated with the gene expressions of their regulators. Co-methylation module-based analyses can greatly reduce the dimension of the data and noise of individual m^6^A peaks. Therefore, we use WGCNA (43) to construct a signed weighted co-methylation network. For each module, we calculate the Pearson correlations between the m^6^A indexes (the first component of principal component analysis) and the expression of 1442 RBPs annotated by GO database respectively. Since Pearson correlation is very sensitive to outliers, we perform Jackknife resampling (leave one out) and take the least significant *P* value for each test (see ‘Materials and Methods’ section for details). We then use random permutation to determine the threshold of significance (see ‘Materials and Methods’ section for details).

On the other hand, if an RBP regulate the m^6^A sites near their targets, we expect to see the co-localization of RBP binding sites with the m^6^A peaks of the module regulated by the RBP, otherwise, the correlation may reflect the indirect effects of regulation such as through regulating the abundance of another m^6^A regulator. In this framework, we integrate the CLIP-seq data of 157 RBPs obtained from starBase (version 3) (48,50) as well as ENCODE CLIP-seq dataset (65) to test whether the RBP binding sites are over-represented in the corresponding m^6^A modules. Since most RBPs do not have available CLIP-seq data, we also take advantage of the RNAcompete-derived motifs (52) as well as several well-known motifs (53–55) of 89 RBPs to test the enrichment of motifs in the associated modules. The RBPs with gene expression significantly correlate with specific modules and with binding targets or motifs significantly enriched in the same modules will be identified as the high-confidence m^6^A regulators specifically regulate the m^6^A sites in the associated modules.

### Systematic analyses of m^6^A methylomes of multiple cell lines revealed credible dynamics of m^6^A

**Figure 2.**
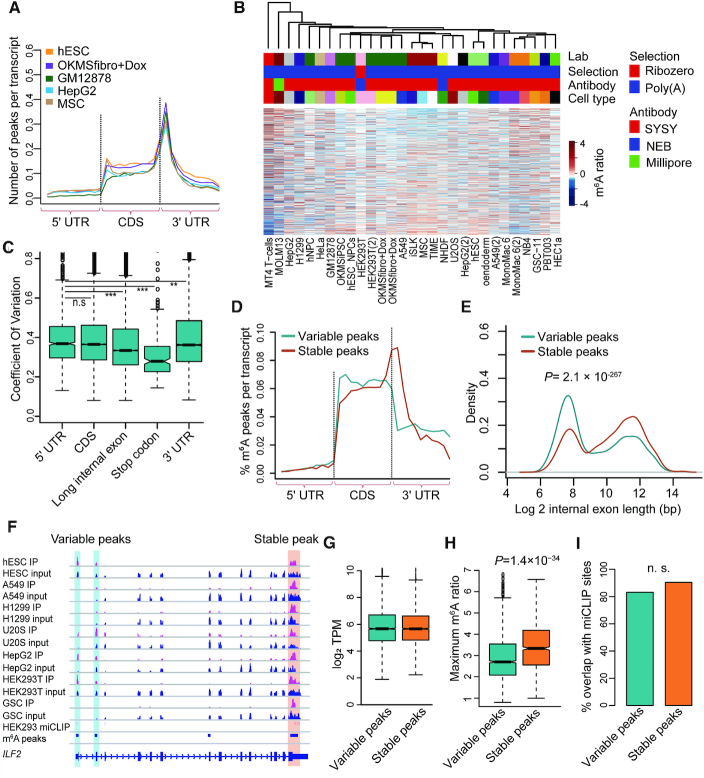
Analyses of m^6^A methylomes of multiple cell lines. (**A**) Normalized distributions of m^6^A peaks across 5′ UTR, CDS and 3′ UTR for representative cell lines. (**B**) The unsupervised hierarchical clustering and heatmap of the m^6^A ratios for the m^6^A peaks with the largest CVs across all cell lines. The technical information is indicated above the heatmap. (**C**) Box plot representing the CVs of m^6^A ratios for the peaks located at different regions of mRNAs. (**D**) Normalized distributions of variable and stable m^6^A peaks across 5′ UTR, CDS and 3′ UTR. (**E**) Densities of logarithm transformed lengths of the internal exons with variable m^6^A peaks and stable m^6^A peaks. The *P*-value of Wilcoxon test is indicated. (**F**) Tracks showing the read coverage of the IPs, inputs and the merged m^6^A peaks of the representative cell lines as well as the HEK293 m^6^A sites from miCLIP-seq data on *ILF2*. The tracks are shown for optimal viewing. The variable and stable m6A peaks are highlighted, respectively. (**G**) Box plots representing the logarithm transformed TPMs of variable and stable m^6^A peaks. (**H**), Box plot representing the maximum m^6^A ratios across all cell lines of variable and stable m^6^A peaks. (**I**) Bar plot showing the percentages of variable peaks and stable peaks that overlap with m^6^A sites obtained from miCLIP-seq. ‘n.s.’ denotes non-significant.

In order to systematically elucidate the cell-specific *trans*-acting regulation of m^6^A using real data, we applied the above computational framework to the public available m^6^A-seq data of 104 samples in 25 unique human cell lines (Supplementary Table S1). We obtained about 15 000 m^6^A peaks for each cell (Supplementary Figure S1A), and the m^6^A peaks for these cell lines were strongly enriched near stop codons, consistent with previous reports (1) (Figure 2A).

To evaluate the reliability of the normalized m^6^A ratios, we performed unsupervised hierarchical clustering using the m^6^A ratios of these samples (replicates were merged) (Figure 2B). We found clear variations of m^6^A ratios and that the samples were not clustered according to the technical issues including labs, RNA selection protocols and antibodies (Figure 2B). Since gene expression obtained from RNA-seq data (input of m^6^A-seq) was less affected by technical bias, we clustered these samples using gene expression to represent the real relationship among these samples (Supplementary Figure S1B). The hierarchical clustering dendrogram generated using m^6^A-seq data were in general similar as that generated using gene expression (*P* = 0.006; permutation test using ‘dendextend’ package (40); Supplementary Figure S1C). The same cell lines (MONO-MAC-6 and HEK293T) from different labs were also clustered together, whereas, the A549 cell lines from different labs were not clustered together possibly due to the diverse expression of m^6^A writers (see Supplementary Text 1 for clarification; Supplementary Figure S1D and E).

### Variable m^6^A sites are away from stop codons

To systematically study the patterns and mechanisms of *trans*-regulating of m^6^A, we combined the m^6^A-seq data of the same cell lines to obtain the methylomes of 25 unique human cell lines. We found different m^6^A peaks had different levels of variations across these cell lines (Supplementary Figure S1F). The m^6^A peaks near stop codons had significantly smaller coefficient of variations (CVs) of m^6^A ratios than the other m^6^A peaks, while the peaks in the long internal exons were slightly smaller than that in UTRs and coding regions (Figure 2C). As shown in Figure 2D, the 11 949 stable m^6^A peaks (CV < 0.3) tended to be enriched near stop codons, whereas the 29 173 variable m^6^A peaks (CV > 0.3) were enriched in coding regions and completely lost the enrichment near stop codons and less enriched in long internal exons, suggesting that the m^6^A sites near stop codons are regulated mainly by *cis*-acting elements rather than *trans*-acting factors (Figure 2E; an example is shown in Figure 2F). Based on this definition, there were about 50% of stable peaks in each cell line (Supplementary Figure S1G), which was consistent with a recent report that *cis*-regulation account for 33–46% of the variability of m^6^A levels (16). In this study, we would like to focus on the variable m^6^A peaks. Compared to those stable m^6^A peaks, the variable m^6^A peaks occurred on the genes with similar gene expression but had significantly smaller maximum m^6^A ratios across all cell lines (Figure 2G and H).

To test whether the variable m^6^A peaks were genuine m^6^A peaks or technical noises, we used single-nucleotide-resolution m^6^A sites in HEK293 cells obtained by miCLIP-seq technology as gold standard to evaluate the m^6^A peaks (42). We found the variable m^6^A peaks and stable m^6^A peaks identified in HEK293T cells exhibited the similar proportions that overlapped with miCLIP-seq reported m^6^A sties, indicating that the variable m^6^A peaks were as genuine as the stable peaks (Figure 2I).

### Modular co-methylation of the variable m^6^A sites revealed prevalent *trans*-acting regulation of m^6^A

**Figure 3.**
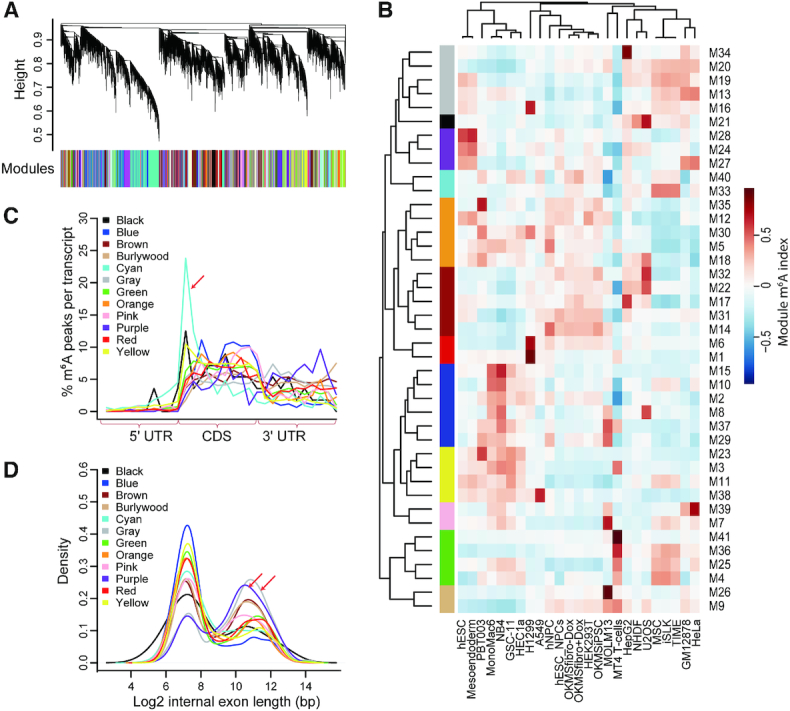
Classification and analyses of co-methylated m^6^A modules. (**A**) Classification of co-methylated m^6^A modules through dynamical cutting of the clustering dendrogram of all variable m^6^A peaks. (**B**) Heatmap representing the m^6^A indexes of all the 41 co-methylation modules across all the cell lines. (**C**) Normalized distributions of m^6^A peaks in different combined modules across 5′ UTR, CDS and 3′ UTR. (**D**) Densities of logarithm transformed lengths of the internal exons with m^6^A peaks in different combined co-methylation modules.

We constructed a signed weighted co-methylation network using WGCNA (43) based on the m^6^A ratios of 29 173 variable m^6^A peaks across the 25 unique cell lines (Figure 3A). We obtained 41 co-methylation modules, which were further merged into 12 larger modules according to the correlations of module m^6^A indexes (the first component of principal component analysis) among them (Figure 3B and Supplementary Table S3). As shown in Figure 3B, the m^6^A indexes of the m^6^A modules showed strong cell type specificities, suggesting that cell-specific m^6^A methylomes may be resulted from co-regulation of m^6^A sites by cell-specific regulators. On the other hand, we found the co-methylation modules showed specific topology of m^6^A and that the m^6^A peaks in one module strongly enriched near translation start sites (Figure 3C), while the m^6^A peaks in another two modules strongly enriched in long internal exons (Figure 3D), suggesting that the topology of m^6^A methylomes are also dynamic and regulated by certain *trans*-acting factors. We also checked the motif enrichment of these modules and found these modules were enriched in distinct motifs (Supplementary Figure S2A). Moreover, the representative module-specific motifs tend to be lowly occurred in other modules, suggesting the different modules are regulated by diverse *trans-*acting factors (Supplementary Figure S2B). The genes in the 12 combined modules were enriched in different GO (47) categories (Supplementary Figure S2C), suggesting that the co-regulated m^6^A sites tend to play specific functional roles in specific cells.

### An m^6^A module was specifically methylated in cancer cell lines

**Figure 4.**
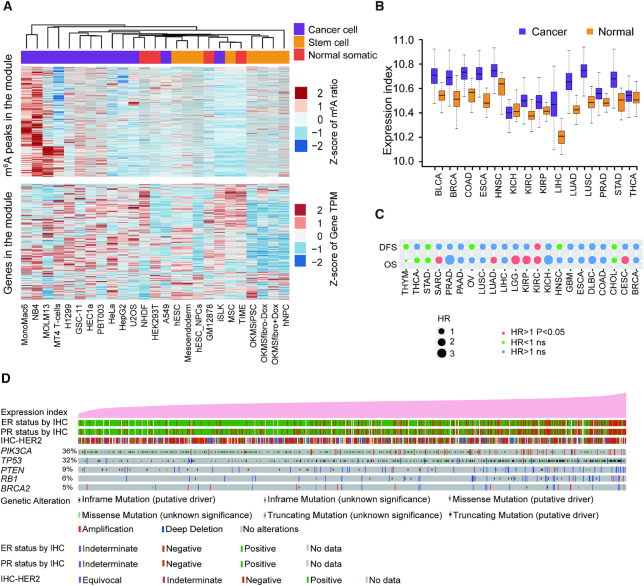
Discovery of a co-methylation module specifically methylated in cancer cell lines. (**A**) Heatmaps representing the *Z*-scores of m^6^A ratios (upper panel) and gene expressions (low panel) of the peaks and corresponding genes across all cell lines. The types of cell lines are indicated at the top of the upper panel. (**B**) Box plot representing the gene expression indexes of the genes corresponding to cancer-specific module for cancer and normal samples of 14 cancer types in TCGA. (**C**) Cox correlations between the gene expression indexes of the genes corresponding to cancer-specific module and the survival of cancer patients of 14 cancer types in TCGA. OS: overall survival; DFS: disease-free survival; HR: hazard ratio. (**D**) Tracks representing the gene expression indexes of the genes corresponding to the cancer-specific module and genetic alteration spectrum of the key markers as well as clinical phenotypes of the breast cancer patients from TCGA. The patient samples are sorted according to the gene expression indexes of the cancer-specific module.

We found one of the 12 combined m^6^A co-methylation modules (blue module) was highly methylated specifically in cancer cell lines other than normal somatic cells as well as stem cells (Figure 4A). As shown in Figure 4A, the expression of the corresponding genes was much higher in those cancer cell lines, suggesting that the enhanced m^6^A methylation at the m^6^A sites in this module may result in elevated abundances of the mRNAs harboring these m^6^As possibly through increasing the RNA stabilities (57). Then, we took advantage of the TCGA (The Cancer Genome Atlas) clinical samples to further address the oncological roles of this module. Interestingly, in 13 of the 14 cancer types included in TCGA, the gene expression indexes of this module were significantly higher in cancer tissues than in normal tissues, suggesting the up-regulation of these genes are common in cancers and may relate to the etiology of most cancers (Figure 4B). Furthermore, the high gene expression indexes of the cancer module were significantly correlated with the shortened survival time of patients in CESC (cervical squamous cell carcinoma), KIRC (kidney renal clear cell carcinoma), KIRP (kidney renal papillary cell carcinoma), LGG (low grade glioma), SARC (sarcoma) and LUAD (lung adenocarcinoma) (Figure 4C and Supplementary Figure S3A–F). In each cancer, we ranked the cancer samples according to the expression indexes of this module to test the correlation between the expression of these genes and genetic alteration spectrum of the key markers as well as clinical phenotypes of these cancers. In breast cancer, the higher expression of these genes was associated with Triple Negative Breast Cancer (TNBC) and positively correlated with the occurrence of somatic mutations of TP53, PIK3CA, PTEN and RB1 (Figure 4D). In LGG, the higher expression of the genes in the module was positively correlated with the occurrence of somatic mutations of TP53, PTEN and EGFR (Supplementary Figure S3G).

### Systematic identification of m^6^A regulators

**Figure 5.**
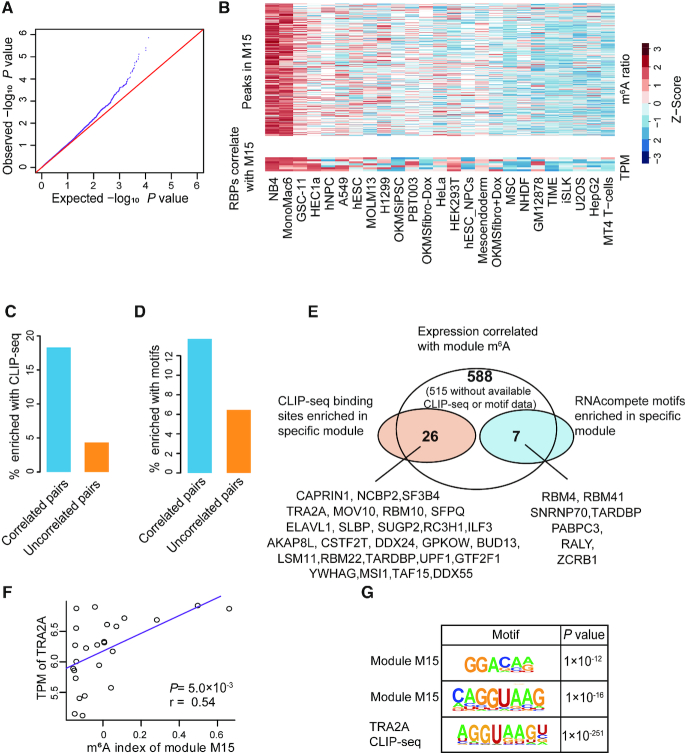
Systematic identification of m^6^A regulators. (**A**) Q-Q plot comparing the distributions of expected *P*-values and observed *P*-values of the correlations between the gene expressions of RBPs and m^6^A indexes of the co-methylation modules. (**B**) Heatmaps representing the m^6^A ratios of the m^6^A peaks within the module M15 (upper panel) and the gene expressions of the RBPs that significantly correlated with the m^6^A indexes of M15 (lower panel). The cell lines are sorted according to the m^6^A indexes of M15. (**C** and**D**) Barplot representing the percentages of the pairs of RBPs and modules that enriched for CLIP-seq binding sites (C) or motifs (D) of the RBPs out of the pairs that showing significant and non-significant (top 1000 least significant) correlations between gene expressions of the RBPs and the m^6^A indexes of the modules. (**E**) Venn diagram demonstrating the identification of 32 high-confidence m^6^A regulators. (**F**) Scatter plot showing the correlation between TRA2A gene expression and m^6^A indexes of module M15 across all cell lines. (**G**) Representative motifs enriched in module M15 and TRA2A CLIP-seq targets.

To systematically identify these regulators that regulated specific m^6^A modules, we used the first component of PCA (Principal Component Analysis) as the m^6^A indexes of the 41 original m^6^A modules. For each module, we calculated the Pearson correlations between the m^6^A indexes and the expression of 1442 RBPs annotated by GO database respectively. As shown in Figure 5A, the *P*-values we observed for all the tests were significantly smaller than the expected *P*-values generated by permutation (see ‘Materials and Methods’ section for details), indicating a significant proportion of real correlations statistically could not be explained by random chances. Based on the profiles of the observed and expected *P*-values, we identified 588 RBPs that were significantly correlated with at least one m^6^A co-methylation module by requiring FDR < 0.2, which denoted less than 118 (20% of 588) RBPs could be identified in random permutations (see Methods for details; Supplementary Figure S4A and B; Table S4). We referred these 588 RBPs to low-confidence m^6^A regulators. As demonstrated in Figure 5B, the gene expression of all the low-confidence m^6^A regulators that correlated with module M15 showed very similar profile as the m^6^A ratios of all the m^6^A peaks in this module (Figure 5B). Since we performed quantile normalization of the m^6^A ratios across all cell lines, as expected, we did not identify METTL3 and METTL14 that may regulate the m^6^A globally. However, we found the gene expressions of RBM15B and ZC3H13, two known components of the writer complex, were positively correlated with module M11 and M25, respectively (Supplementary Figure S4C and D), while the expression of m^6^A eraser ALKBH5 was negatively correlated with module M14 (Supplementary Figure S4E), suggesting that some components of writer complex as well as demethylase may also confer specificities of m^6^A. Besides, we also found a previously reported specific m^6^A regulator SMAD3, which can specifically promote the installation of m^6^A (20), was positively correlated with module M33 (Supplementary Figure S4F). The proteins of m^6^A regulators would possibly interplay with m^6^A writers or erasers at their binding sites on RNAs, we collected the published Mass Spectrum data of protein pull-down using the antibodies of METTL3, METTL14, WTAP, VIRMA (4,59,66), we found 108 RBPs out of the 588 low-confidence m^6^A regulators could be pulled down by at least one of the antibodies (Supplementary Figure S4G). On the other hand, we found 44 RBPs out of the low-confidence m^6^A regulators could be pulled down by m^6^A modified oligos (58) (Supplementary Figure S4H).

Then we took advantage of the CLIP-seq data of 157 RBPs obtained from starBase (version 3) (48,50) as well as ENCODE CLIP-seq dataset (65) to test whether the RBP binding sites were over-represented in the corresponding m^6^A modules. There were 22% of the significantly correlated pairs of RBP expression and m^6^A module showed significant enrichment of CLIP-seq peaks in the same m^6^A modules, in contrast, it was only 4% for the top 1000 least significant pairs of RBP and m^6^A modules, indicating a significantly enriched occurrences of co-localization of RBP binding sites with their significantly correlated modules (*P* = 5.8 × 10^−8^, two-tailed Chi-square test; Figure 5C). Since most RBPs did not have available CLIP-seq data, we utilized the RNAcompete-derived motifs (52) as well as several well-known motifs (53–55) of 89 RBPs for further evaluation. Similarly, we observed a trend that the motifs of the RBPs were more likely to show significant enrichment in the m^6^A modules correlated with the RBPs as compared to the uncorrelated modules (*P* = 0.09, two-tailed Chi-square test; Figure 5D). The RBP motifs were mostly enriched within 50bp of m^6^A motifs, suggesting that the RBPs may tend to regulate the m^6^A sites around 50bp of its binding sites (Supplementary Figure S4I). In the end, out of the 50 and 26 low-confidence m^6^A regulators with available CLIP-seq data and known motifs respectively, 26 (52%) and 7 (27%) RBPs also showed significant co-localization with the exact correlated m^6^A modules based on CLIP-seq and motif analyses respectively (Figure 5E and Supplementary Table S4). We referred these 32 RBPs to high-confidence m^6^A regulators. As exemplified, the gene expression of an RBP TRA2A significantly correlated with the m^6^A ratio of module M15 (Figure 5F), which happened to enrich for a motif resembled the TRA2A motif obtained from CLIP-seq (Figure 5G). On the other hand, since there were 516 low-confidence m^6^A regulators without available CLIP-seq datum or known motif, we would expect a dramatic increase of the high-confidence m^6^A regulators when more and more CLIP-seq data become available in the future.

### Experimental validations of selected m^6^A regulators

Because eCLIP-seq of plenty of RBPs had been performed in HepG2 cells (65), we selected 3 high-confidence m^6^A regulators TRA2A, CAPRIN1 and MOV10, which were highly expressed and with corresponding module highly methylated in HepG2 cells, to experimentally validate their regulatory functions on m^6^A. First of all, we tested whether knocking down of these regulators affected the stoichiometry of some m^6^A peaks in human HepG2 cells using low-input m^6^A-seq (64). The m^6^A peaks identified in low-input m^6^A-seq were enriched in stop codons as expected (Supplementary Figure S5A). Then we performed Co-Immunoprecipitation (Co-IP) to test whether these regulators interacted with the major m^6^A writers (METTL3 and METTL14) and erasers (FTO and ALKBH5).

**Figure 6.**
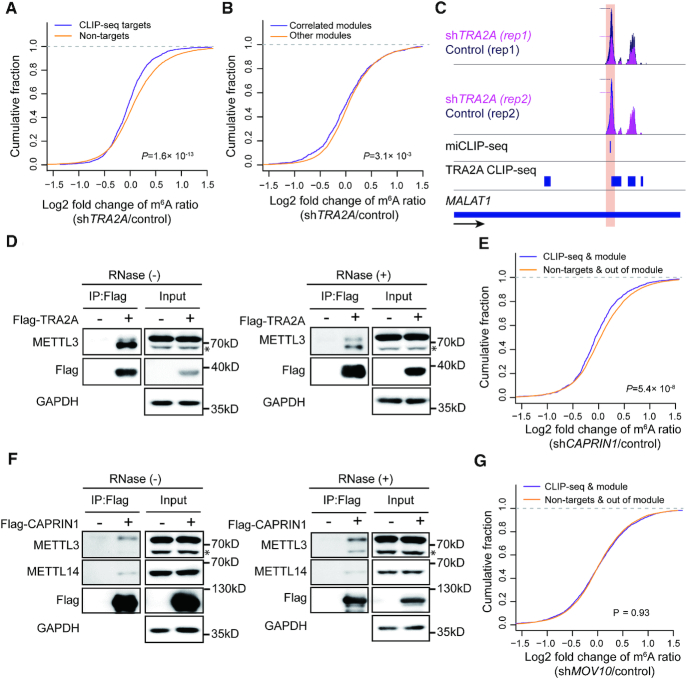
Experimental validation of selected m^6^A regulators. (**A**) Plot of cumulative fraction of log2 fold change of m^6^A ratios upon *TRA2A* knockdown for the m^6^A peaks overlap or non-overlap with TRA2A CLIP-seq targets. *P*-value of two-tailed Wilcoxon test is indicated. (**B**) Plot of cumulative fraction of log2 fold change of m^6^A ratios upon *TRA2A* knockdown for the m^6^A peaks within or not within the co-methylation modules correlated with TRA2A. *P* value of two-tailed Wilcoxon test is indicated. (**C**) Tracks displaying the read coverage of IPs normalized by inputs as well as the miCLIP-seq m^6^A sites and TRA2A CLIP-seq peaks in HepG2 cells on the long non-coding gene *MALAT1*. The m^6^A peak with down-regulated m^6^A ratio in sh*TRA2A* is highlighted. The dashed lines indicate the summits of the peaks. (**D**) Western blots showing the interaction between TRA2A and METTL3 with and without RNase treatment respectively. * indicates a non-specific band (see Supplementary Figure S5B). (**E**) Plot of cumulative fraction of log2 fold change of m^6^A ratios upon *CAPRIN1* knockdown comparing the m^6^A peaks in the correlated modules and overlap with CAPRIN1 CLIP-seq targets versus the peaks not within the correlated module or overlap with CAPRIN1 CLIP-seq targets. *P*-value of two-tailed Wilcoxon test is indicated. (**F**) Western blots showing the interactions of CAPRIN1 with METTL3 and METTL14 with and without RNase treatment respectively. * indicates a non-specific band (see Supplementary Figure S5B). (**G**) Plot of cumulative fraction of log2 fold change of m^6^A ratios upon *MOV10* knockdown comparing the m^6^A peaks in the correlated modules and overlap with MOV10 CLIP-seq targets versus the peaks not within the correlated module or overlap with MOV10 CLIP-seq targets. *P*-value of two-tailed Wilcoxon test is indicated.

The m^6^A ratios of the m^6^A peaks with TRA2A binding were significantly down-regulated upon TRA2A depletion as compared to the m^6^A peaks without TRA2A binding (*P* = 1.6 × 10^−13^, two-tailed Wilcoxon test; Figure 6A), indicating that TRA2A promoted the installation of m^6^A through binding near the m^6^A sites other than indirect effects such as regulating another m^6^A regulator. Similarly, the m^6^A ratios of the predicted m^6^A module regulated by TRA2A were also significantly down-regulated upon TRA2A depletion as compared to other modules (*P* = 3.1 × 10^−3^, two-tailed Wilcoxon test; Figure 6B). The above results were very consistent with our observation that the expression of TRA2A was positively correlated with the m^6^A indexes of the corresponding modules. As exemplified in Figure 6C, the TRA2A bound m^6^A peak in the long non-coding RNA *MALAT1* was downregulated upon *TRA2A* depletion. We further found TRA2A interacted with METTL3 independent of RNAs, suggesting that TRA2A promote the installing of m^6^A near its binding sites through recruitment of METTL3 (Figure 6D and Supplementary Figure S5B).

Similar results were observed for CAPRIN1, the m^6^A ratios of the m^6^A peaks within the related module and co-localized with CAPRIN1 were significantly down-regulated upon *CAPRIN1* depletion as compared to the m^6^A peaks in other modules and without CAPRIN1 binding (*P* = 5.4 × 10^−8^, two-tailed Wilcoxon test; Figure 6E), though the CAPRIN1 CLIP-seq data were obtained from a different cell line. Strikingly, we found CAPRIN1 interacted with both METTL3 and METTL14, suggesting that CAPRIN1 can recruit the methyltransferase complex to promote the m^6^A installation near its binding sites (Figure 6F). Whereas, we did not find the depletion of MOV10 changed the m^6^A ratios of the peaks within the related module (Figure 6G), nor did we find the interaction of MOV10 with any of the m^6^A writers or erasers. Therefore, MOV10 was not like a genuine m^6^A regulator.

Therefore, we finally validated TRA2A and CAPRIN1 as genuine m^6^A regulators, and MOV10 was a false positive discovery. We further identified 427 and 124 differentially methylated m^6^A peaks due to knockdown of *TRA2A* and *CAPRIN1* respectively using exomePeak software (60). Similar as the CLIP-seq binding targets of these RBPs, the differentially methylated m^6^A peaks were all enriched in protein coding regions other than near stop codons, consistent with our finding that m^6^A peaks near stop codons were stable (Supplementary Figure S5C and D).

We then tried to investigate whether these m^6^A regulators had any functional consequences by regulating m^6^A. We noticed that *TRA2A* knockdown resulted in upregulated gene expression of 470 genes and downregulated gene expression of only 79 genes, the up-regulated genes were significantly enriched in KEGG pathway related to protein processing in endoplasmic reticulum as well as metabolism (Supplementary Figure S6A). Of the 470 upregulated genes, there were 107 genes with at least one m^6^A peak co-localized with TRA2A CLIP-seq peak, these genes were enriched in the pathway of protein processing in endoplasmic reticulum, suggesting the functional role of TRA2A on homeostasis of endoplasmic reticulum by regulation m^6^A. Since m^6^A was reported to promote the degradation of RNAs (8), we hypothesized that TRA2A induced the m^6^A modification of specific RNAs to facilitate their degradations. To test this hypothesis, we selected 11 upregulated genes with multiple m^6^A sites co-localized with TRA2A CLIP-seq binding sites to examine the effects of TRA2A on their RNA stabilities. We found 7 of the 11 genes showed significantly increased stability in *TRA2A* knockdown HepG2 cells, including 3 genes (*HSPA8*, *RRBP1*, *UGGT1*) involved in ‘protein processing in endoplasmic reticulum’ (Supplementary Figure S6B–D). After cultured for several generations, we also noticed remarkably decreased viability of *TRA2A* knockdown HepG2 cells based on colony formation assay (Supplementary Figure S6E), which was possibly due to the induction of endoplasmic reticulum stress in the longtime culturing of cells with defects in maintaining homeostasis of endoplasmic reticulum.

## DISCUSSION

In this study, we successfully developed a computational framework to systematically identify *trans* regulators of m^6^A through integrating gene expressions, binding targets, and binding motifs of a large number of RBPs with a co-methylation network constructed using large-scale m^6^A methylomes across diverse cell states. Applying the framework to the public available m^6^A-seq data of 25 unique cell lines revealed pervasive *trans*-acting regulation of m^6^A and identified 32 high-confidence m^6^A regulators with reasonable experimental validation rate.

The successful identification of m^6^A regulators using 25 distinct cell lines demonstrated a powerful and widely portable strategy to elucidate the *trans*-acting regulation of m^6^A based on a batch of m^6^A methylomes. In this study, we definitely underestimated the prevalence of m^6^A regulators due to technical limitations, such as the limited number of cell lines, lack of available CLIP-seq data for most RBPs. Moreover, we probably also missed the m^6^A regulators that played important roles in extremely specific cells or physiological and pathological processes. Since m^6^A-seq technology becomes more and more applicable and affordable (64), large-scale m^6^A-seq data in specific biological systems will be available in near future. It is of great advantage to apply our computational framework to these data in order to uncover the *trans*-acting mechanisms that may be important for specific systems. For example, applying the framework to a population of cancer samples may reveal novel m^6^A regulators specifically regulate specific m^6^A sites involved in tumorigenesis in certain types of cancers.

We noticed that there were two types of m^6^A sites according to their variation among multiple cell lines. It is interesting that the m^6^As around stop codons tend to be hard wired thus very stable among different cells lines, these m^6^A sites could be considered as indispensable ‘structural m^6^A sites’. They are installed at specific positions and are important for the basic functions and biogenesis of mRNAs. The structural m^6^A sites around stop codons are probably regulated mainly in *cis* and directly mediated by m^6^A methyltransferase complex. This idea is supported by the previous report that VIRMA and ZC3H13, which are important components of methyltransferase complex, specifically deposit m^6^A around the stop codon of mRNA (4,5). On the other hand, the m^6^A sites away from stop codons, such as those within coding regions, tend to display cell-specificities, thus could be considered as ‘dynamic m^6^A sites’. They are precisely and dynamically regulated through a number of regulators expressed with spatial and temporal specificities, providing a novel mechanism for genes to play diverse roles in different cells. As previously reported, transcription factor CEBPZ induces the m^6^A specifically within the coding region of its associated mRNAs through co-transcriptional recruitment of METTL3 at the promoters (19). In this study, we found TRA2A and CAPRIN1 also selectively modulate the m^6^A sites within the coding regions (Supplementary Figure S5C and D). These results further support that dynamic m^6^A sites are away from stop codons.

Our study provided a new perspective on how the m^6^A sites were regulated. It is well known that m^6^As are modified co-transcriptionally (17,67), thus m^6^A can be regulated through co-transcriptional mechanisms. Two well-known m^6^A regulators SMAD2/3 and CEBPZ are both transcription factors and regulate m^6^A co-transcriptionally (19,20). Moreover, a recent study reported that H3K36me3, a histone marker for transcription elongation, could guide the installation of m^6^A modifications with classic enrichment near stop codons through direct recruitment of METTL14 (18). In this study, besides transcription factors, we also found classic RBPs worked as regulators that selectively regulated subsets of m^6^A sites through direct recruitments of methyltransferase complex, suggesting that various RBPs and transcription factors work together to modulate the precise levels of specific m^6^A sites. In contrast to transcription factors, which always bind to the promoters, the RBPs confer the m^6^A specificity by their RNA binding specificities. Moreover, the m^6^A sites can be controlled precisely through the modulations of multiple regulators. Therefore, it is very likely that m^6^A RNA methylation is precisely controlled in a similar manner as alternative splicing, which is regulated complicatedly by histone modifications co-transcriptionally as well as a variety of splicing factors that bind the *cis*-regulatory elements of splicing (68).

Technically, reliable quantification of m^6^A sites on mRNAs is still of great challenges. Therefore, we made multiple methodological improvements in order to mitigate the impacts of technical biases of m^6^A-seq data, which were important for the successful identification of m^6^A regulators. First of all, we compared our winscore-based method with exomePeak (60) and MeTPeak (69) using one of the HepG2 m^6^A-seq dataset. Though our winscore-based peaks had a similar number and distribution across 5′UTR, CDS and 3′UTR as exomePeak and MeTPeak, the density of m^6^A motifs (number of RRAC motifs in 100 bp of peak) of winscore-based peaks were more than 2-fold higher than exomePeak and MeTPeak called peaks (Supplementary Figure S7A and B), suggesting our peaks are more centralized to real m^6^A sites. This should be important for the quantification of m^6^A peaks, because non-centralized long peaks may dilute the signals of m^6^A differences. Another important technical detail was that we defined the m^6^A ratio of each merge peaks with multiple sliding windows as the maximum m^6^A ratios of all windows for each sample, respectively. The exact locations and widths of m^6^A peaks may be biased by the RNA fragment lengths, sequencing read lengths and *et al.*, it is of great advantage to use the peak summits of each library for the overlapped peaks other than the exact same region when comparing the m^6^A ratios using m^6^A-seq data from different labs. Third, to calculate the m^6^A ratios, we required the input RPKM > 5 to deal with the unreliable m^6^A ratios with low read coverage. Fourth, we used quantile normalization to normalize the m^6^A ratios across all samples, thus the bias caused by different immunoprecipitation efficiencies across the libraries were minimized. Last but the most important, we identified the m^6^A regulators based on m^6^A modules other than single m^6^A sites, which greatly minimized the impact of using the noisy m^6^A quantifications of single m^6^A peaks.

## DATA AVAILABILITY

The raw data of the low-input m^6^A-seq data have been deposited in the Sequence Read Archive (SRA) database (https://dataview.ncbi.nlm.nih.gov/) under the accession number SRP211943.

## Supplementary Material

gkz1206_Supplemental_Files
