# Supplementary material for: Integrative network analysis identifies cell-specific trans regulators of m6A
Source: Nucleic Acids Res. 2020 Jan 8;48(4):1715–29. doi: 10.1093/nar/gkz1206 (PMC7038928; doi:10.1093/nar/gkz1206)

Text 1

We found the A549 cell lines from two different labs showed similar gene expression but less correlation of m^6A, when we checked the gene expression of m^6A writers, we realized gene expression of *METTL3 and WTAP* tended to be higher in the second lab, indicating that the A549 cells from two different labs did have diverse m^6A methylomes (Figure S1D and E). This perhaps reflected the more sensitive cellular response of m^6A machinery upon uncontrolled environmental stimulation in cell culture.

There were HepG2 cell lines from two different lab, the gene expression of these two cell lines were not clustered together, suggesting that the two cell lines indeed have diverse transcriptomes.

Figure S1. Analyses of m^6A methylomes.

(A) Barplot representing the number of m^6A peaks identified in each cell line. The error bars denote the maximum and minimum across all replicates. **(B)** The unsupervised hierarchical clustering and heatmap of the TPMs for the genes with the largest CVs across all cell lines. The technical information is indicated above the heatmap. **(C)** Comparison of the cluster dendrograms generated using TPMs (left) and m^6A ratios (right). **(D)** Barplot representing the TPMs of m^6A writers in the A549 cell lines from two different labs. **(E)** Scatter plot comparing the TPMs of all genes in the A549 cell lines from two different labs. The METTL14 and WTAP are indicated. **(F)** Histogram of the CVs of m^6A ratios of all m^6A peaks. **(G)** Stacked barplot representing the fraction of stable and variable m^6A peaks in all the unique cell lines.

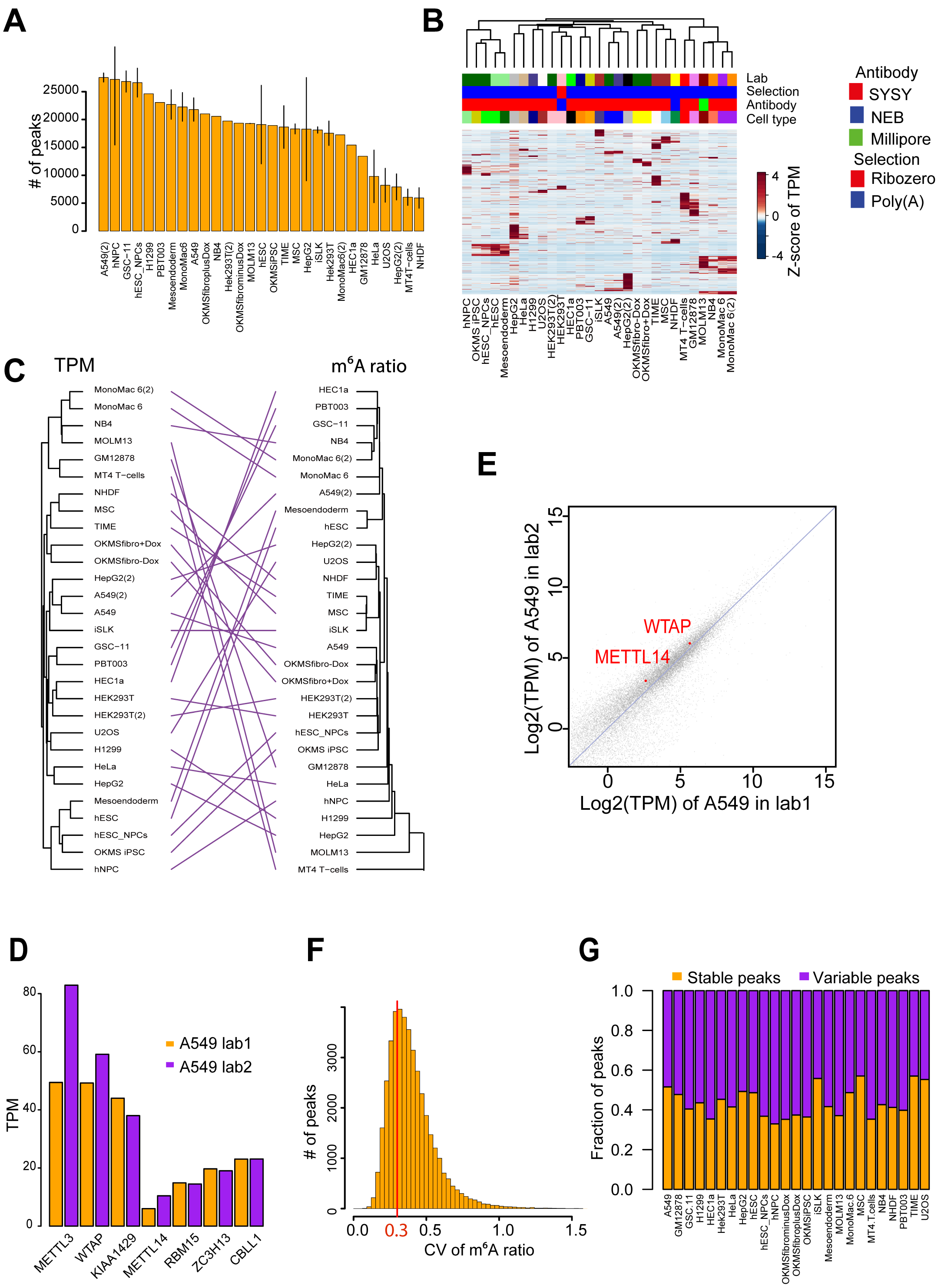

A
# of peaks
25000
20000
15000
10000
5000
0
A549(2)
hNPC
GSC-11
hESC_NPCs
H1299
PBT003
Mesoendoderm
MonoMac6
A549
OKMSfibroplusDox
NB4
Hek293T(2)
OKMSfibrominusDox
MOLM13
hESC
OKMSiPSC
TIME
MSC
HepG2
iSLK
Hek293T
MonoMac6(2)
HEC1a
GM12878
HeLa
U2OS
HepG2(2)
MT4T-cells
NHDF
B
Lab
Selection
Antibody
Cell type
Antibody
SYSY
NEB
Millipore
Selection
Ribozero
Poly(A)
4
0
-4
Z-score of TPM
hNPC
OKMS iPSC
hESC_NPCs
hESC
Mesoendoderm
HepG2
HeLa
H1299
U2OS
HEK293T(2)
HEK293T
HEC1a
PBT003
GSC-11
iSLK
A549
A549(2)
HepG2(2)
OKMSfibro-Dox
OKMSfibro+Dox
TIME
MSC
NHDF
MT4 T-cells
GM12878
MOLM13
NB4
MonoMac 6
MonoMac 6(2)
C
TPM
m6A ratio
MonoMac 6(2)
MonoMac 6
NB4
MOLM13
GM12878
MT4 T-cells
NHDF
MSC
TIME
OKMSfibro+Dox
OKMSfibro-Dox
HepG2(2)
A549(2)
A549
iSLK
GSC-11
PBT003
HEC1a
HEK293T
HEK293T(2)
U2OS
H1299
HeLa
HepG2
Mesoendoderm
hESC
hESC_NPCs
OKMS iPSC
hNPC
HEC1a
PBT003
GSC-11
NB4
MonoMac 6(2)
MonoMac 6
A549(2)
Mesoendoderm
hESC
HepG2(2)
U2OS
NHDF
TIME
MSC
iSLK
A549
OKMSfibro-Dox
OKMSfibro+Dox
HEK293T(2)
HEK293T
hESC_NPCs
OKMS iPSC
GM12878
HeLa
hNPC
H1299
HepG2
MOLM13
MT4 T-cells
E
Log2(TPM) of A549 in lab2
Log2(TPM) of A549 in lab1
0
5
10
15
WTAP
METTL14
D
TPM
80
60
40
20
0
A549 lab1
A549 lab2
METTL3
WTAP
KIAA1429
METTL14
RBM15
ZC3H13
CBLL1
F
# of peaks
3000
2000
1000
0
0.0
0.3
0.5
1.0
1.5
CV of m6A ratio
G
Stable peaks
Variable peaks
Fraction of peaks
1.0
0.8
0.6
0.4
0.2
0.0
A549
GM12878
GSC.11
H1299
HEC1a
Hek293T
HeLa
HepG2
hESC
hESC_NPCs
hNPC
OKMSfibrominusDox
OKMSfibroplusDox
OKMSiPSC
iSLK
Mesoendoderm
MOLM13
MonoMac.6
MSC
MT4.T.cells
NB4
NHDF
PBT003
TIME
U2OS

Figure S2. Motif and GO analyses of co-methylated m^{6}A modules.

(A) Enriched motifs of representative co-methylation modules. **(B)** Radar plot representing the percentages of peaks that contain the representative motifs enriched in 6 different modules out of the 6 modules. The colors of lines indicate the modules. *P* value of two-tailed Chi-square test is indicated at the bottom. **(C)** GO analysis of each co-methylation module.

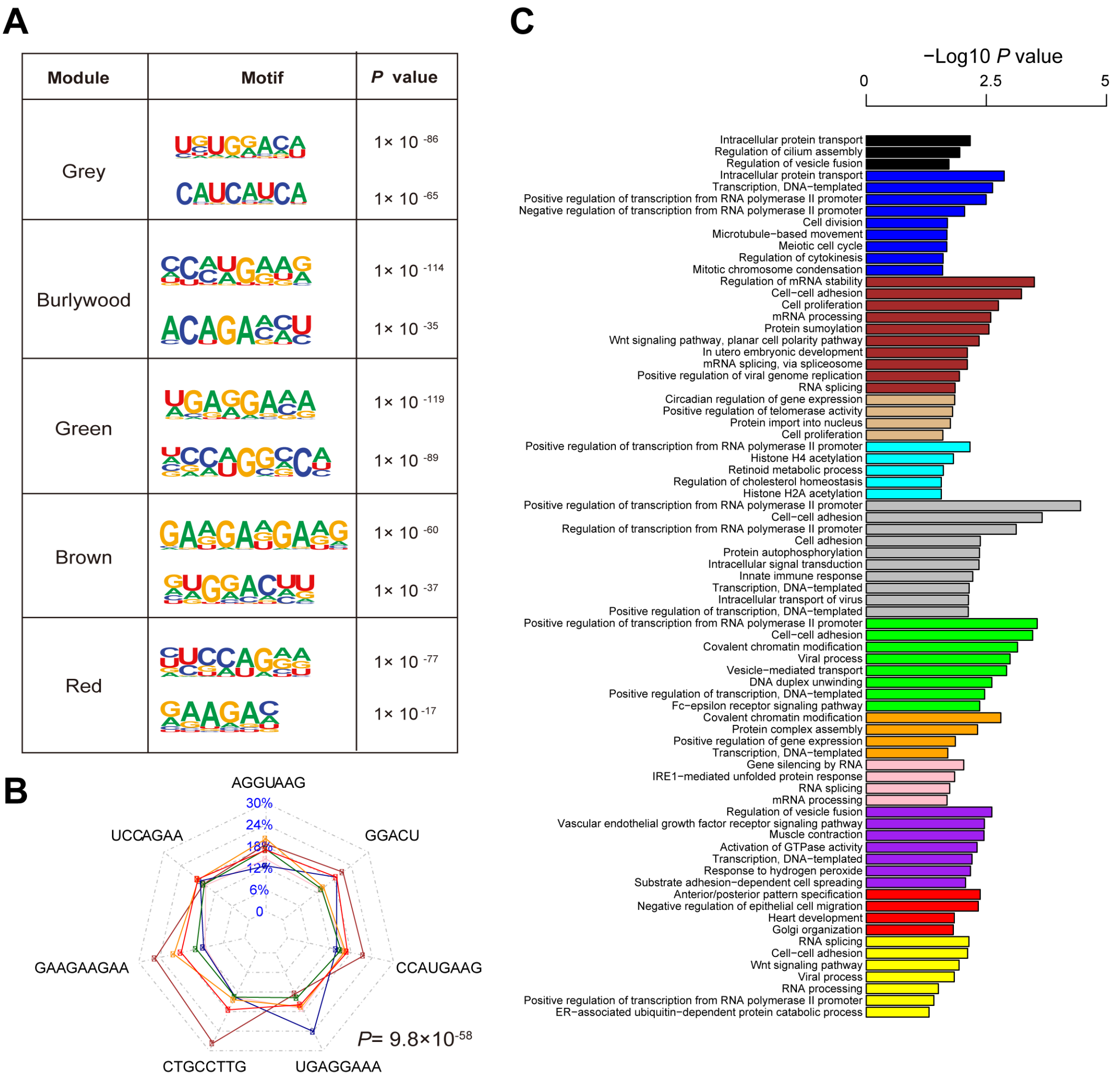

Figure S3. TCGA analyses of the cancer-specific module.

(A-F) Kaplan-Meier survival plots representing the correlations between the gene expression indexes of the cancer-specific module and the overall survival of SARC **(A),** CESC **(B)**, KIRP **(C)**, KIRC **(D)**, and LGG **(E)** as well as the disease-free survival of KIRC **(F)**. OS: overall survival; DFS: disease-free survival; HR: hazard ratio. **(G)** Tracks representing the gene expression indexes of the cancer-specific module and genetic alteration spectrum of the key markers as well as clinical phenotypes of the LGG from TCGA. The patient samples are sorted according to the gene expression indexes of the cancer-specific module.

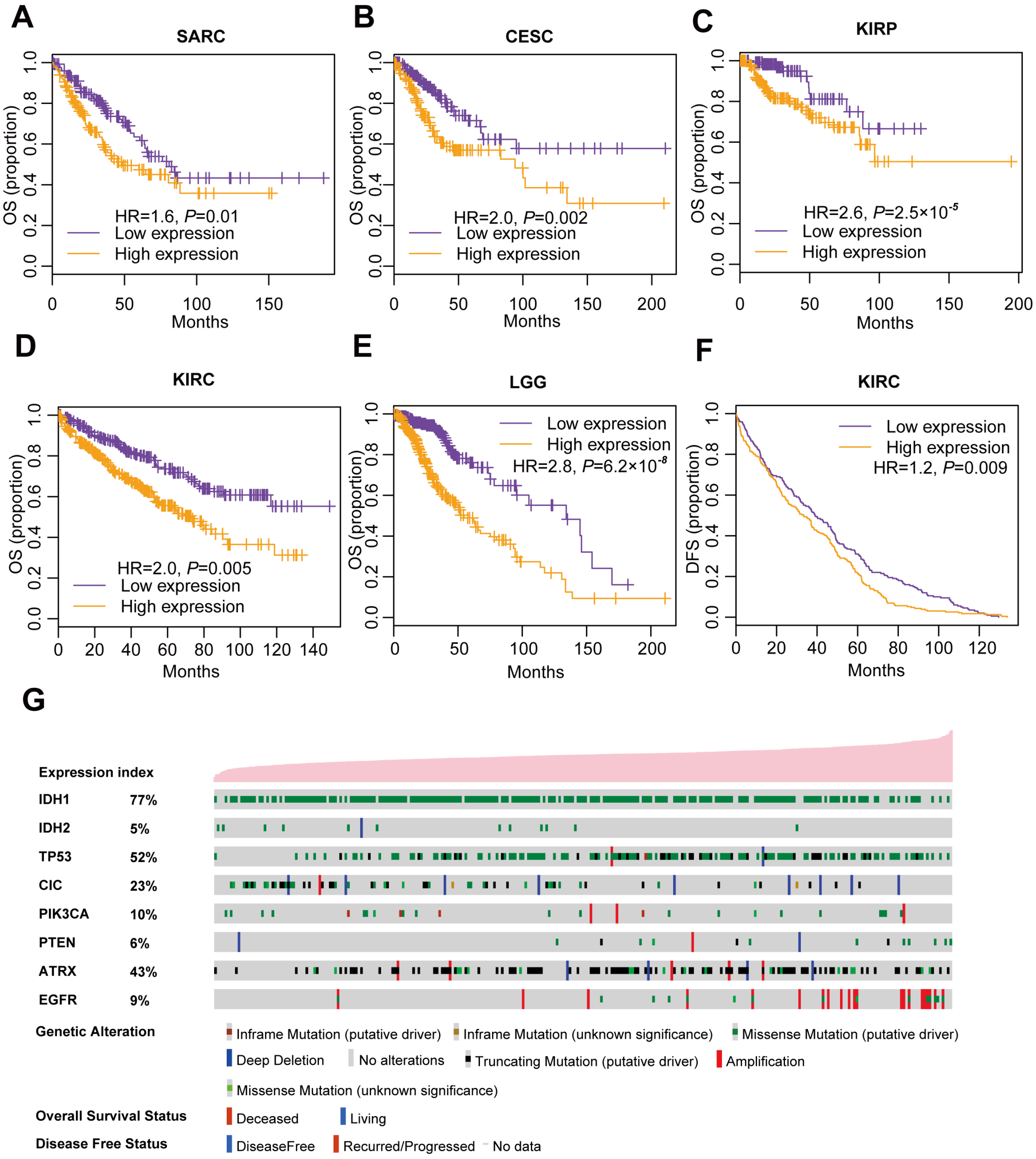

Figure S4. Identification of m^{6}A regulators

(A) Determination of *P* value cutoff to obtain FDR of 0.2 according to the permutations. **(B)** Plot displaying the RBPs that correlate with each module with FDR < 0.2. **(C-F)** Scatter plot showing the correlations of m^{6}A indexes of specific modules with the gene expressions of RBM15B (C), ZC3H13 (D), ALKBH5 (E) and SMAD3 (F) across all cell lines. **(G)** Stacked barplot representing the percentages of low-confidence regulators in the IP-MS using m^{6}A writers. Numbers of proteins are indicated in the middles of the bars. **(H)** Venn diagram representing the overlap of low-confidence m^{6}A regulators and IP-MS obtained proteins using m^{6}A modified oligos. **(I)** Density plots showing the distances of specific RBP motifs to the m^{6}A motif GGACU.

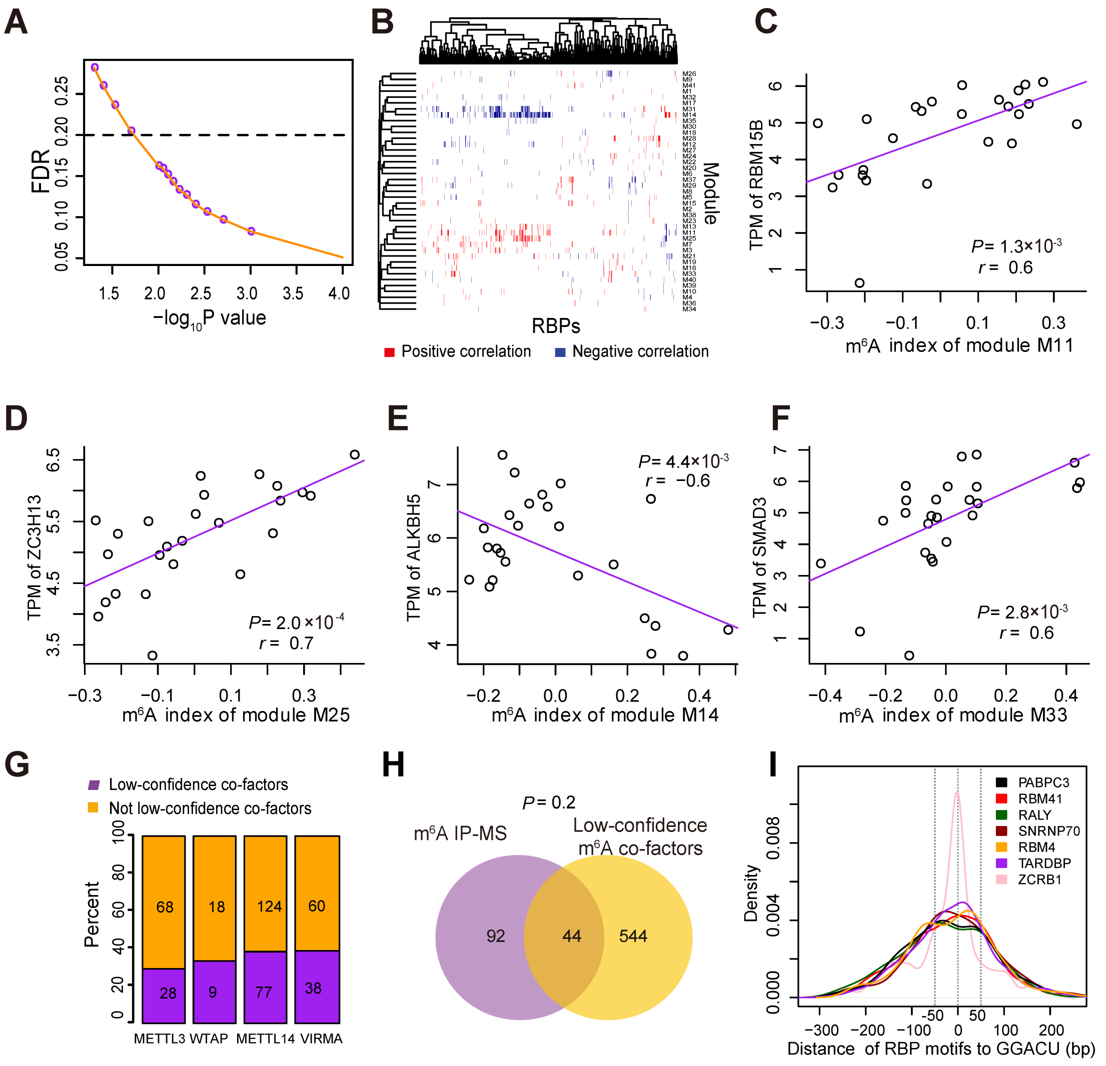

Figure S5. Experimental validations of m^{6}A regulators.

(A) Normalized distributions of m^{6}A peaks across 5' UTR, CDS, and 3' UTR for all samples. **(B)** Western blot showing the lower band of METTL3 is a non-specific band based on *METTL3* knockout. **(C-D)** Normalized distributions of differential m^{6}A peaks up RBP knockdown and CLIP-seq targets of TRA2A **(C)** and CAPRIN1 **(D)**, across 5' UTR, CDS, and 3' UTR.

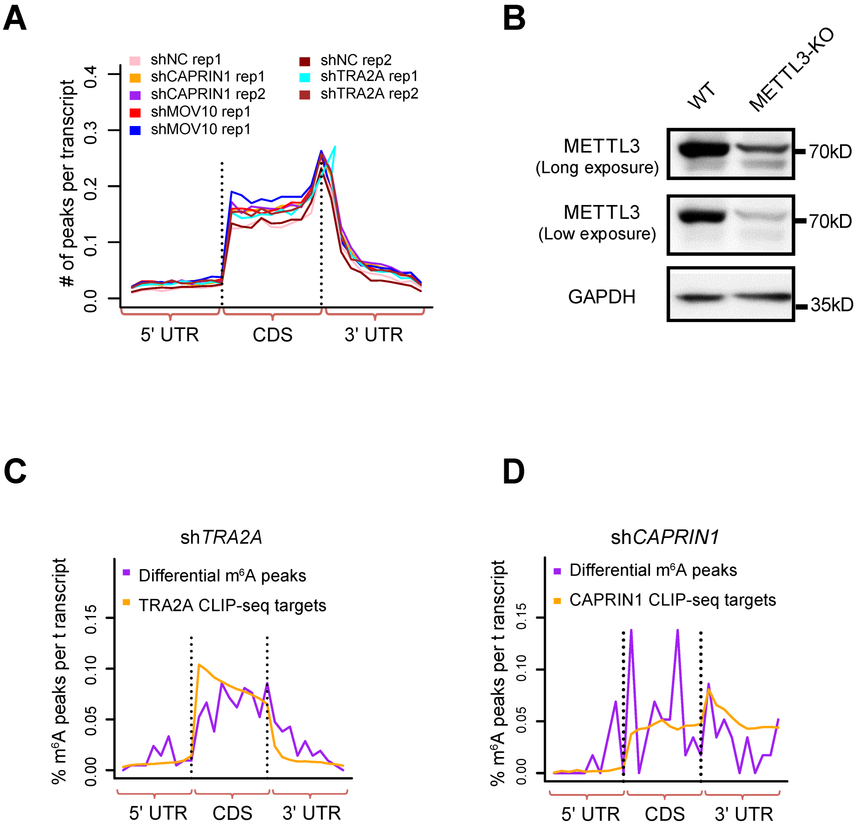

Figure S6. Knock-down of *TRA2A* affects RNA stability.

(**A**) KEGG pathway analysis of up-regulated genes after *TRA2A* knockdown. (**B-D**) Representative mRNA profile of *HSPA8*, *RRBP1*, *UGGT1* at 0-, 2-, and 4-h time points after actinomycin D (ActD, 5 μg/ml) treatment. Error bars represent mean standard deviations of 2 replicates. **P* < 0.05, ***P* < 0.01, ****P* < 0.001, Student's t-test. (**E**) Effect of *TRA2A* silencing on colony formation ability.

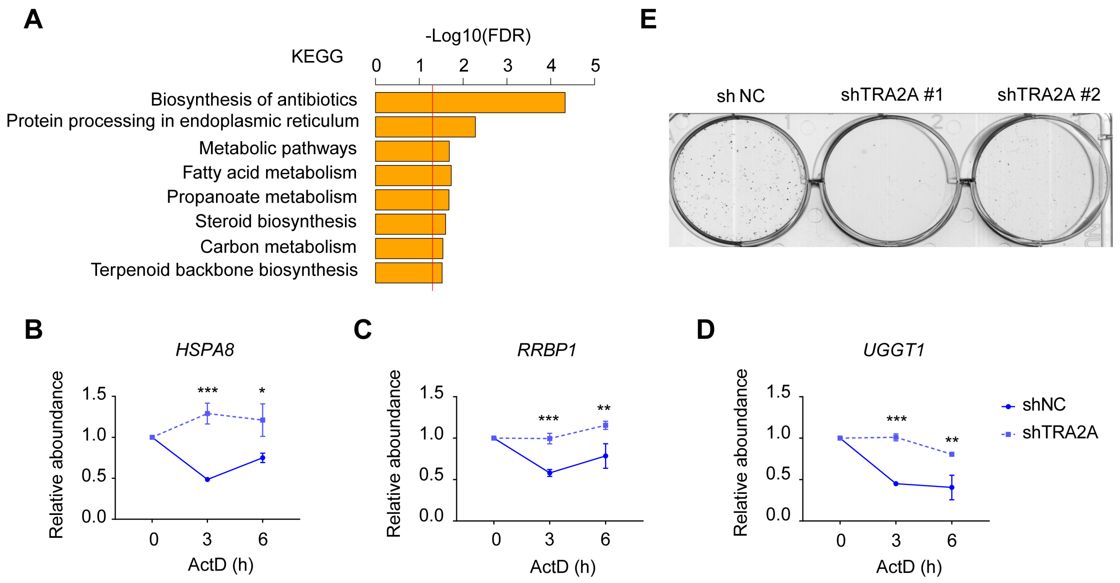

Figure S7. Comparing the winscore-based m^{6}A peaks with exomePeak and MeTPeak identified peaks. (A) Normalized distributions of m^{6}A peaks across 5' UTR, CDS, and 3' UTR for the peaks identified using different tools. The numbers of the peaks are indicated in the parentheses. (B) Barplot comparing the densities of m^{6}A motifs of the m^{6}A peaks identified using different tools.

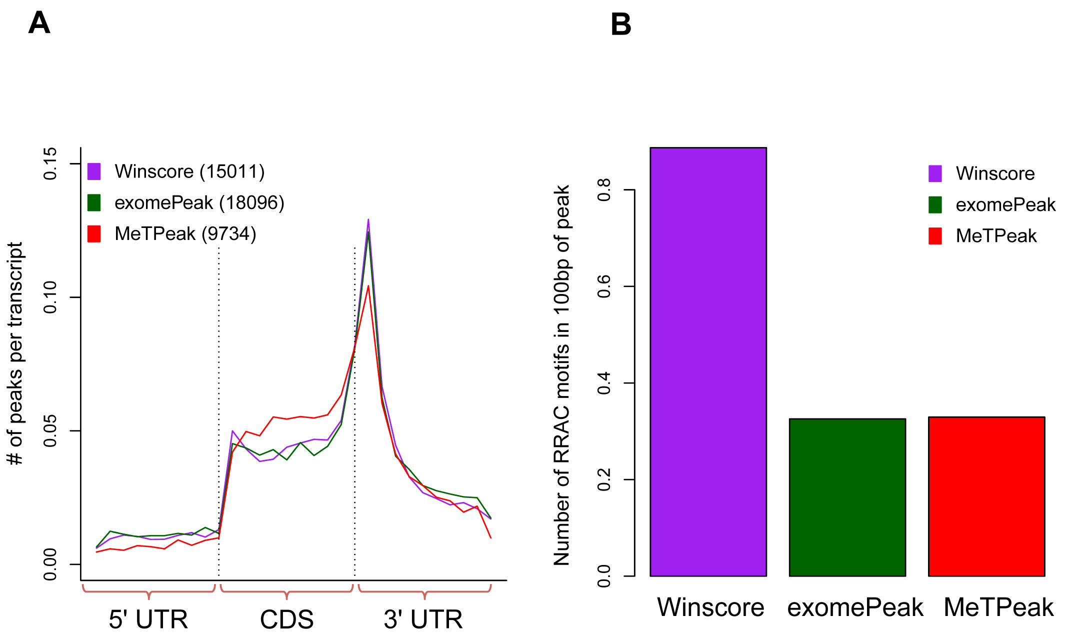

Supplement: gkz1206_Supplemental_Files [file gkz1206_supplemental_files.zip › Supplementary Figures clean.pdf]
